# Availability of healthcare resources and epithelial ovarian cancer stage of diagnosis and mortality among Blacks and Whites

**DOI:** 10.1186/s13048-017-0352-1

**Published:** 2017-08-22

**Authors:** Swati Sakhuja, Huifeng Yun, Maria Pisu, Tomi Akinyemiju

**Affiliations:** 1 Department of Epidemiology, University of Alabama at Birmingham, Birmingham, Alabama USA; 2 Comprehensive Cancer Center, University of Alabama at Birmingham, Birmingham, Alabama USA; 3 Division of Preventive Medicine, University of Alabama at Birmingham, Birmingham, Alabama USA; 4 Department of Epidemiology, University of Kentucky, Lexington, Kentucky USA

**Keywords:** Racial disparities, Ovarian cancer, Survival, Healthcare

## Abstract

**Background:**

The purpose of this study is to examine whether racial disparities in epithelial ovarian cancer stage at diagnosis and survival may be explained by geographic availability of healthcare resources among Blacks and Whites.

**Methods:**

Data from the Surveillance, Epidemiology and End Results (SEER) database was used to identify White and Black women ages 40 years and above diagnosed with epithelial ovarian cancer between 2000 and 2010. Data on county-level availability of healthcare resources was obtained from the Area Resource File. Multi-level regression models, overall and stratified by race and age, were used to examine the associations of health care access (HCA) and socioeconomic status (SES) with stage at diagnosis while Cox proportional hazards models were used to examine the association with survival.

**Results:**

Among 46,423 women diagnosed with epithelial ovarian cancer, Blacks were more likely to reside in counties with fewer average number of oncology hospitals (*p* < 0.05) and hospitals with ultrasound (*p* < 0.001), but higher number of medical doctors (*p* < 0.0001) and Ob/Gyn (*p* < 0.001). Black patients had higher odds of late stage diagnosis of epithelial ovarian cancer (OR: 1.13, 95% CI: 1.04–1.25) and higher risk of epithelial ovarian cancer mortality (HR: 1.25, 95% CI: 1.19–1.32) compared with White patients after accounting for differential availability of healthcare resources. Among Black patients, residing in counties with fewer medical doctors was associated with increased odds of late stage diagnosis (OR: 1.86, 95% CI: 1.10–3.13), and the racial disparity in late stage diagnosis and mortality was larger among patients ages <65 years compared with older patients.

**Conclusion:**

Racial disparities in availability and utilization of healthcare resources likely contributes to adverse epithelial ovarian cancer outcomes among Black women in the US.

## Background

Ovarian cancer is the leading cause of death from gynecological malignancies in the US [1, 2] and the fifth most common cause of death among all cancer sites. [3, 4] There are over 22,000 incident cases and 15,000 deaths annually in the US, making it one of the most lethal cancers for women. [3] Prognosis of ovarian cancer mainly depends on stage at diagnosis, however screening for ovarian cancer has not been proven to be an effective method for early detection. [5–7] Persistent racial, socio-economic (SES) and healthcare access (HCA) disparities exist in ovarian cancer outcomes, [8–10] suggesting that there may be differential access to potentially life-saving treatment strategies [11].

Studies examining disparities in ovarian cancer outcomes have observed that while SES is significantly associated with mortality, it does not fully explain racial disparities in receipt of treatment or mortality. [12, 13] Beyond SES, access to healthcare is another critical predictor of cancer treatment and survival. Healthcare access comprehensively defined incorporates aspects of availability, affordability, accommodation, acceptability and accessibility. [14] Disparities in these components are also influenced by other socio-cultural factors, and even in equal access settings such as the Veteran’s Administration, racial disparities in accessibility and utilization of cancer care have been documented, [15–17] with Blacks more likely to experience barriers to care and worse health outcomes [17].

Geographic access to healthcare and utilization of care in relation to cancer outcomes is a relatively understudied and highly complex area of disparities research. It is also an essential factor for early diagnosis and receipt of high quality and timely treatment, elements critical for the survival of women with ovarian cancer. [11, 18] Prior studies have shown that the availability of healthcare resources has significant influence on health outcomes, including breast and colorectal cancer survival. [19–21] The influence of healthcare availability on health outcomes also appears to vary by race; a higher number of medical personnel and health facilities in US counties was observed not to be significantly associated with reduced late stage presentation and longer survival for Black colorectal cancer patients, but was associated with improved outcomes for White colorectal cancer patients. [19, 21] Since Black women are more likely to present with late stage of disease and have higher mortality, despite lower ovarian cancer incidence compared with Whites, [22–25] examining if and how availability of healthcare resources influences ovarian cancer stage and mortality among Blacks and Whites may improve understanding of the causes of persistent racial disparities, while highlighting potential areas for targeted intervention strategies.

In this paper, we examine racial disparities in availability of healthcare resources and association with epithelial ovarian cancer stage at diagnosis and survival among Blacks and Whites in the US.

## Methods

*Data Source and Study population:* Data for this study was obtained from the National Cancer Institute Surveillance, Epidemiology and End Results (SEER) program using the SEER 18 Registries (excluding AK and Louisiana), Nov 2012 Submission - linked To County Attributes - Total U.S., 1969–2011 Counties plus Area Resource File (ARF-2008). SEER covers about 28% of the U.S. population, and includes detailed information for each cancer diagnosis on demographics, disease stage, histology, surgery of primary site, vital status and survival months. Eligibility criteria for this study includes White and Black adult women ages 40 years and above with a primary diagnosis of epithelial ovarian cancer represented in the registry and diagnosed between 2000 and 2010.

*Outcome Variables:* There were two primary outcomes of interest in this study: 1) Stage at diagnosis (excluding in situ cases), defined as late stage (if distant) or early stage (if localized or regional) using SEER summary stage 2000 (1998+) variable; and 2) Survival defined as time in months from diagnosis to death due to epithelial ovarian cancer as identified in the SEER database.

*Health Care Availability (HCA):* Data on county level healthcare resources was obtained from the Area Health Resource File (AHRF) developed by the U.S Health Resources and Services Administration. Details can be obtained from: http://ahrf.hrsa.gov. We defined HCA using the following variables for the year 2005 per million population: number of hospitals, number of hospitals with oncology services, number of hospitals with ultrasound services, number of Obstetricians/Gynecologists (OB/GYN), and number of physicians. The percent of individuals who have no health insurance was also included in the analysis, as this may influence the concentration of hospitals, clinics or medical personnel in particular neighborhoods. County level data was utilized for this study since decisions on the location of healthcare resources such as hospitals and clinics often are often made at the county and/or municipal level.

*Study Covariates:* County level SES was included in the analysis and defined based on*:* percent of population with less than a 9th grade education and percent of families living below the federal poverty level for the year 2000. Individual level data on histologic type using International Classification of Diseases for Oncology, Third Edition (ICD-O-3) codes [26]) and primary site-specific surgery were also included in the analysis as dichotomous variables based on information in the SEER database. The analysis focused on epithelial ovarian cancer, and other less common subtypes were excluded for analysis based on ICD-O-3 codes (i.e., 8000–8004, 8240–8245, 8590–8670, 8800–8951 and 9060–9975).

*Statistical analysis*: We compared the distribution of socio-demographic, clinical, histologic and county-level HCA by race/ethnicity using chi-Square tests for categorical variables and analysis of variance test (ANOVA) for continuous variables. We first examined whether racial differences existed between Blacks and Whites in epithelial ovarian cancer stage at diagnosis and survival after adjusting for demographic, clinical, HCA and SES variables. We formally assessed for effect modification by including interaction terms for race and HCA, and age and HCA. Next, we conducted race- and age- stratified analysis to determine whether HCA variables were associated with stage or survival in both racial groups and by age-group. This allowed us to examine whether racial disparities existed in epithelial ovarian cancer outcomes among women <65 years (who are more likely to have private insurance and Medicaid) and those >65 years (who are eligible for Medicare insurance). To examine racial differences in epithelial ovarian cancer stage, we conducted consecutive multilevel regression model which allowed us to include random effects into the model thereby accounting for between-county variation and within-county correlation. To examine racial differences in epithelial ovarian cancer survival, we examined time to ovarian cancer-related deaths using Cox proportional hazards models adjusting for both individual level (demographics, clinical) and county level (HCA, SES) variables and accounted for clustering. Patients were censored at the time of death, or end of follow-up (December 2013). Further, we assessed proportionality of hazards by plotting survival curves and using a time dependent predictor in the hazards model. All statistical analyses were conducted using SAS version 9.4.

## Results

There were 46,423 Black and White women with epithelial ovarian cancer diagnosed from 2000 to 2010 in the SEER registry. Among these patients, 93.1% were White and 6.9% were Black (Table 1). Blacks were more likely to be diagnosed with epithelial ovarian cancer at a younger age compared with Whites (Blacks: 27.2%, Whites: 25.2% ages 40–54, *p*-value: < 0.0001). Blacks were less likely to undergo site-specific surgery for epithelial ovarian cancer as compared to Whites (61.0% vs. 77.0%, *p*-value: <0.0001). Black patients were more likely to live in counties with greater number of medical doctors (1101 vs. 934, *p*-value: < 0.0001), counties with greater number of Ob/Gyn (157 vs. 127, *p*-value: < 0.0001), but counties with fewer numbers of hospitals with ultrasound machines (9.6 vs. 10.8, *p*-value: < 0.0001) compared with Whites. In addition, Black patients were more likely to reside in counties with a higher proportion of families living below the poverty level (43.0% vs. 22.8%, *p*-value: < 0.0001) compared with White patients.

**Table 1:** Socio-demographic and healthcare access characteristics of epithelial ovarian cancer patients by race/ethnicity, SEER 2000–2010

Characteristics	Blacks 3219 (6.90%)	Whites 43,204 (93.07%)	*P*-value
Demographics and clinical characteristics			
Age at Diagnosis (years)			
40–54	876 (27.21)	10,865 (25.15)	**<0.0001**
55–64	847 (26.31)	10,923 (25.28)	
65–74	762 (23.67)	9746 (22.56)	
75+	734 (22.80)	11,670 (27.01)	
Late Stage			
Yes	2483 (75.74)	30,322 (70.18)	**<0.0001**
No	781 (24.26)	12,882 (29.82)	
Site-specific Surgery			
Yes	1963 (60.98)	33,253 (76.97)	**<0.0001**
No	1227 (38.12)	9676 (22.40)	
Unknown	29 (0.90)	275 (0.64)	
Death^a^			**<0.0001**
Yes	1574 (58.96)	19,355 (50.72)	
No	1051 (40.04)	18,803 (49.28)	
County-Level Healthcare Availability			
% Uninsured			
Mean (SD)	15.91 (4.07)	15.17 (4.84)	**<0.0001**
< 25th percentile	439 (13.64)	10,813 (25.03)	**<0.0001**
25-75th percentile	1994 (61.94)	21,688 (50.21)	
> 75th percentile	786 (24.40)	10,697 (24.76)	
^b^Hospitals			
Mean (SD)	16.56 (13.57)	16.80 (17.69)	0.6545
< 25th percentile	403 (12.52)	8782 (20.33)	**<0.0001**
25-75th percentile	1909 (59.03)	23,590 (54.61)	
> 75th percentile	907 (28.18)	10,826 (25.06)	
^b^Oncology			
Mean (SD)	7.00 (5.40)	7.32 (10.25)	**0.0078**
< 25th percentile	910 (28.27)	12,920 (29.91)	**<0.0001**
25-75th percentile	962 (29.89)	18,343 (42.46)	
> 75th percentile	1347 (41.85)	11,935 (27.63)	
^b^Medical Doctors			
Mean (SD)	1101.16 (545.33)	934.27 (557.10)	**<0.0001**
< 25th percentile	499 (15.50)	10,727 (24.83)	**<0.0001**
25-75th percentile	1641 (50.98)	21,873 (50.63)	
> 75th percentile	1079 (33.52)	10,598 (24.53)	
^b^OB/GYN			
Mean (SD)	157.29 (84.84)	127.51 (69.43)	**<0.0001**
< 25th percentile	422 (13.11)	10,572 (24.47)	**<0.0001**
25-75th percentile	1663 (51.66)	22,808 (52.80)	
> 75th percentile	1134 (35.23)	9818 (22.73)	
^b^Hospitals with Ultrasound			
Mean (SD)	9.56 (11.36)	10.81 (15.54)	**<0.0001**
< 25th percentile	625 (19.42)	9502 (22.00)	**<0.0001**
25-75th percentile	1890 (58.71)	23,262 (53.85)	
> 75th percentile	704 (21.87)	10,434 (24.15)	
County-Level Socioeconomic Status			
% Less than 9th Grade Education			
Mean (SD)	8.56 (4.26)	8.40 (4.86)	**0.0209**
< 25th percentile	355 (11.03)	11,461 (26.53)	**<0.0001**
25-75th percentile	2053 (63.78)	21,040 (48.71)	
> 75th percentile	811 (25.19)	10,697 (24.76)	
% Families below poverty level			
Mean (SD)	10.62 (4.31)	8.77 (4.50)	**<0.0001**
< 25th percentile	438 (13.61)	11,232 (26.00)	**<0.0001**
25-75th percentile	1398 (43.43)	22,110 (51.18)	
> 75th percentile	1383 (42.96)	9856 (22.82)	

^a^Ovarian cancer specific deaths only

^b^per million population

Late stage defines as distant metastasis at presentation

Bold indicates significance (*p* value <0.05)

In multivariable adjusted models with HCA variables (Table 2), Blacks had 13% higher odds for late stage diagnosis of epithelial ovarian cancer (OR: 1.13, 95% CI: 1.04–1.25) compared with Whites. In the model adjusting for both HCA and SES, Blacks remained at 14% higher odds of late stage diagnosis (OR: 1.14, 95% CI: 1.04–1.25) and the interaction between race and number of medical doctors in the county was statistically significant (*p* value = 0.0354). Additionally, epithelial ovarian cancer mortality was 25% higher among Blacks compared with Whites in adjusted models with HCA variables (HR: 1.25, 95% CI: 1.19–1.32) as well as HCA and SES adjusted model (HR: 1.24, 95% CI: 1.18–1.31). Residing in counties with fewer medical doctors was independently associated with increased risk for mortality (HR: 1.12, 95% CI: 1.04–1.20) and this association remained after additionally adjusting for SES (HR: 1.11, 95% CI: 1.03–1.20). Further, there was also a significant linear trend in the association between in-county number of medical doctors and epithelial ovarian cancer mortality (*p* value: <0.0001).

**Table 2:** Multivariable adjusted analysis for late-stage epithelial ovarian cancer diagnosis and mortality, SEER 2000–2010

	Late Stage Diagnosis AOR (95% CI)		Mortality AHR (95% CI)^c^	
	Odds Ratio		Hazard Ratio	
	HCA Only^a^	HCA and SES^b^	HCA Only^a^	HCA and SES^b^
Race				
Blacks	**1.13 (1.04–1.25)**	**1.14 (1.04–1.25)**	**1.25 (1.19–1.32)**	**1.24 (1.18–1.31)**
Whites	Ref	Ref	Ref	Ref
Healthcare Availability				
% Uninsured				
< 25th percentile	0.98 (0.89–1.08)	1.02 (0.93–1.13)	0.95 (0.91–1.00)	1.00 (0.94–1.07)
25 – 75th percentile	1.06 (0.93–1.08)	1.02 (0.93–1.13)	**0.94 (0.91–0.98)**	0.98 (0.93–1.04)
> 75th percentile	Ref	Ref	Ref	Ref
# Hospitals				
< 25th percentile	1.01 (0.92–1.11)	1.04 (0.94–1.14)	0.95 (0.89–1.00)	0.96 (0.90–1.02)
25 – 75th percentile	1.00 (0.92–1.08)	0.99 (0.91–1.07)	0.99 (0.94–1.04)	1.00 (0.95–1.05)
> 75th percentile	Ref	Ref	Ref	Ref
# Oncology Hospitals				
< 25th percentile	0.95 (0.87–1.03)	0.98 (0.90–1.07)	0.95 (0.91–1.00)	0.96 (0.91–1.02)
25 – 75th percentile	0.98 (0.92–1.05)	0.99 (0.93–1.06)	0.97 (0.93–1.01)	0.97 (0.93–1.01)
> 75th percentile	Ref	Ref	Ref	Ref
# Medical Doctors^d^				
< 25th percentile	1.10 (0.97–1.25)	1.10 (0.97–1.25)	**1.12 (1.04–1.20)**	**1.11 (1.03–1.20)**
25 – 75th percentile	1.07 (0.97–1.18)	1.07 (0.97–1.18)	**1.06 (1.002–1.12)**	1.05 (0.99–1.12)
> 75th percentile	Ref	Ref	Ref	Ref
# OB/GYN				
< 25th percentile	**0.86 (0.76–0.98)**	**0.84 (0.74–0.95)**	1.01 (0.94–1.09)	1.01 (0.93–1.09)
25-75th percentile	**0.89 (0.81–0.99)**	**0.87 (0.78–0.96)**	1.01 (0.95–1.07)	1.01 (0.95–1.08)
> 75th percentile	Ref	Ref	Ref	Ref
# Hospitals with Ultrasound				
< 25th percentile	1.05 (0.94–1.17)	1.02 (0.92–1.14)	1.00 (0.94–1.07)	1.00 (0.94–1.07)
25-75th percentile	0.99 (0.91–1.08)	0.99 (0.91–1.08)	0.97 (0.92–1.02)	0.96 (0.91–1.01)
> 75th percentile	Ref	Ref	Ref	Ref

^a^Adjusted for county level HCA variables, age, surgery of primary site and seer registry

^b^Adjusted for county level HCA and SES variables, age, surgery of primary site and seer registry

^c^Additionally adjusted for late stage diagnosis

^d^Linear trend test with ovarian cancer mortality was significant at *p* < 0.0001 level

# Number per million population

Among Black patients (Table 3), residing in counties with fewer medical doctors was associated with 86% increased odds of late stage diagnosis in the HCA only model (OR: 1.86, 95% CI: 1.10–3.13) and almost doubled the odds in the HCA and SES model (OR: 1.91, 95% CI: 1.13–3.24) compared with residing in the highest quartile. There were no significant associations between HCA and odds of late stage diagnosis among Whites. There were also no significant associations with epithelial ovarian cancer mortality in the HCA and SES models for Blacks. However, Whites residing in counties with fewer medical doctors experienced a 12% increased risk of epithelial ovarian cancer mortality in the HCA only (HR: 1.12, 95% CI: 1.04–1.21) and HCA and SES (HR: 1.12, 95% CI: 1.03–1.21) models (Table 4).

**Table 3:** Multivariable adjusted odds ratios for late-stage epithelial ovarian cancer diagnosis by race, SEER 2000–2010

	Blacks AOR (95% CI)		Whites AOR (95% CI)		p-interaction^c^
	HCA Only^a^	HCA and SES^b^	HCA Only^a^	HCA and SES^b^	
Healthcare Availability					
% Uninsured					0.1014
< 25th percentile	0.76 (0.753–1.08)	0.82 (0.48–1.40)	1.00 (0.94–1.09)	1.02 (0.88–1.17)	
25 – 75th percentile	1.03 (0.79–1.34)	0.99 (0.66–1.49)	1.01 (0.94–1.09)	1.03 (0.93–1.14)	
> 75th percentile (Ref)	Ref	Ref	Ref	Ref	
# Hospitals					0.7025
< 25th percentile	1.08 (0.71–1.63)	1.23 (0.78–1.92)	1.01 (0.92–1.11)	1.03 (0.93–1.14)	
25 – 75th percentile	1.14 (0.82–1.60)	1.15 (0.82–1.61)	0.99 (0.91–1.08)	0.98 (0.90–1.06)	
> 75th percentile (Ref)	Ref	Ref	Ref	Ref	
# Oncology Hospitals					0.6876
< 25th percentile	0.90 (0.64–1.26)	0.97 (0.68–1.39)	0.95 (0.87–1.04)	0.97 (0.89–1.07)	
25 – 75th percentile	1.13 (0.87–1.48)	1.25 (0.93–1.68)	0.97 (0.91–1.04)	0.98 (0.91–1.05)	
> 75th percentile (Ref)	Ref	Ref	Ref	Ref	
# Medical Doctors					**0.0354**
< 25th percentile	**1.86 (1.10–3.13)**	**1.91 (1.13–3.24)**	1.06 (0.93–1.21)	1.06 (0.93–1.21)	
25 – 75th percentile	**1.53 (1.10–2.12)**	**1.57 (1.12–2.19)**	1.03 (0.93–1.14)	1.03 (0.93–1.15)	
> 75th percentile (Ref)	Ref	Ref	Ref	Ref	
# OB/GYN					0.3754
< 25th percentile	0.67 (0.40–1.13)	0.61 (0.35–1.05)	0.88 (0.78–1.00)	**0.86 (0.75–0.98)**	
25-75th percentile	**0.68 (0.49–0.95)**	**0.62 (0.43–0.88)**	0.91 (0.82–1.01)	**0.89 (0.80–0.99)**	
> 75th percentile (Ref)	Ref	Ref	Ref	Ref	
# Hospitals with Ultrasound					0.7730
< 25th percentile	1.05 (0.69–1.60)	1.05 (0.68–1.61)	1.05 (0.94–1.17)	1.03 (0.92–1.15)	
25-75th percentile	0.94 (0.68–1.28)	0.94 (0.68–1.29)	0.99 (0.90–1.08)	0.99 (0.91–1.09)	
> 75th percentile (Ref)	Ref	Ref	Ref	Ref	

^a^Adjusted for age, surgery of primary site and seer registry

^b^Additionally adjusted for SES variables

^c^For interaction terms between race and each HCA variable

# Number per million population

Bold indicates significance (*p* value ≤0.05)

**Table 4:** Multivariable adjusted hazards ratios for epithelial ovarian cancer mortality by race, SEER 2000–2010

	Blacks AHR (95% CI)		Whites AHR (95% CI)		p-interaction^c^
	HCA Only^a^	HCA and SES^b^	HCA Only^a^	HCA and SES^b^	
Healthcare Availability					
% Uninsured					0.3326
< 25th percentile	1.12 (0.90–1.38)	1.24 (0.91–1.68)	**0.95 (0.90–0.99)**	1.00 (0.93–1.07)	
25 – 75th percentile	1.11 (0.95–1.29)	1.08 (0.86–1.35)	**0.94 (0.90–0.97)**	0.98 (0.93–1.03)	
> 75th percentile (Ref)	Ref	Ref	Ref	Ref	
# Hospitals					0.1093
< 25th percentile	**0.73 (0.57–0.94)**	0.83 (0.63–1.08)	0.96 (0.90–1.02)	0.97 (0.91–1.03)	
25 – 75th percentile	0.94 (0.77–1.14)	0.95 (0.78–1.16)	0.99 (0.94–1.05)	1.00 (0.95–1.05)	
> 75th percentile (Ref)	Ref	Ref	Ref	Ref	
# Oncology Hospitals					0.5587
< 25th percentile	0.92 (0.76–1.12)	0.94 (0.76–1.16)	0.96 (0.91–1.01)	0.97 (0.91–1.02)	
25 – 75th percentile	1.12 (0.96–1.31)	1.13 (0.95–1.34)	0.96 (0.92–1.00)	0.97 (0.93–1.01)	
> 75th percentile (Ref)	Ref	Ref	Ref	Ref	
# Medical Doctors					0.8371
< 25th percentile	1.20 (0.88–1.63)	1.18 (0.87–1.61)	**1.12 (1.04–1.21)**	**1.12 (1.03–1.21)**	
25 – 75th percentile	1.02 (0.84–1.24)	1.04 (0.85–1.26)	**1.07 (1.01–1.14)**	1.06 (0.997–1.13)	
> 75th percentile (Ref)	Ref	Ref	Ref	Ref	
# OB/GYN					0.8834
< 25th percentile	1.02 (0.75–1.39)	1.00 (0.72–1.38)	1.01 (0.93–1.08)	1.00 (0.92–1.09)	
25-75th percentile	1.08 (0.89–1.31)	1.04 (0.85–1.27)	1.00 (0.94–1.07)	1.01 (0.94–1.08)	
> 75th percentile (Ref)	Ref	Ref	Ref	Ref	
# Hospitals with Ultrasound					0.5357
< 25th percentile	1.06 (0.83–1.36)	1.05 (0.82–1.35)	1.00 (0.93–1.07)	0.99 (0.93–1.07)	
25-75th percentile	0.95 (0.79–1.13)	0.94 (0.79–1.13)	0.97 (0.92–1.02)	0.96 (0.91–1.01)	
> 75th percentile (Ref)	Ref	Ref	Ref	Ref	

^a^Adjusted for age, surgery of primary site, late stage diagnosis and seer registry

^b^Additionally adjusted for county level SES variables

^c^For interaction terms between race and each HCA variable

# Number per million population

Bold indicates significance (*p* value ≤0.05)

Among patients <65 years (Table 5), Blacks had a 20% higher odds of late stage diagnosis compared with Whites (OR: 1.20, 95% CI: 1.07–1.35), however, there was no racial difference in stage at diagnosis observed among patients ages 65 years and older. Although, women 65 years and older residing in county with fewer medical doctors had higher odds for late stage diagnosis compared to those living in county with higher number of medical doctors (OR: 1.29, 95% CI: 1.06–1.58), this association was reversed for those residing in counties with fewer number of Ob/Gyn (OR: 0.74, 95% CI: 0.61–0.90). Blacks had higher risk for epithelial ovarian cancer mortality than Whites in both age groups (<65 years HR: 1.27, 95% CI: 1.17–1.37) and (65 years and older HR: 1.14, 95% CI: 1.06–1.22), although the disparity was larger in the <65 years age group.

**Table 5:** Age-stratified multivariable adjusted analysis for late-stage epithelial ovarian cancer diagnosis and mortality, SEER 2000–2010

	Late Stage Diagnosis AOR (95% CI)		p-interaction^b^	Mortality AHR (95% CI)^a^		p-interaction^c^
	<65 years	> = 65 years		<65 years	> = 65 years	
Race						
Blacks	**1.20 (1.07–1.35)**	1.00 (0.87–1.15)		**1.27 (1.17–1.37)**	**1.14 (1.06–1.22)**	
Whites	Ref	Ref		Ref	Ref	
Healthcare Availability						
% Uninsured			0.5071			0.6687
< 25th percentile	0.96 (0.82–1.13)	0.96 (0.78–1.18)		1.04 (0.94–1.16)	0.99 (0.91–1.08)	
25 – 75th percentile	1.01 (0.90–1.13)	1.02 (0.88–1.18)		1.02 (0.94–1.11)	0.97 (0.90–1.03)	
> 75th percentile	Ref	Ref		Ref	Ref	
# Hospitals			0.8476			0.2352
< 25th percentile	1.11 (0.98–1.26)	0.94 (0.81–1.10)		0.99 (0.90–1.10)	0.92 (0.85–1.00)	
25 – 75th percentile	1.03 (0.93–1.14)	0.93 (0.82–1.06)		0.97 (0.90–1.05)	1.01 (0.95–1.07)	
> 75th percentile	Ref	Ref		Ref	Ref	
# Oncology Hospitals			0.4025			0.4809
< 25th percentile	0.97 (0.87–1.08)	1.01 (0.89–1.16)		0.98 (0.90–1.06)	0.94 (0.87–1.00)	
25 – 75th percentile	0.99 (0.91–1.08)	1.03 (0.92–1.14)		0.95 (0.89–1.01)	0.99 (0.94–1.05)	
> 75th percentile	Ref	Ref		Ref	Ref	
# Medical Doctors			0.3598			0.8026
< 25th percentile	1.00 (0.86–1.17)	**1.29 (1.06–1.58)**		**1.15 (1.03–1.30)**	1.06 (0.96–1.17)	
25 – 75th percentile	0.98 (0.87–1.11)	**1.24 (1.07–1.45)**		**1.12 (1.02–1.23)**	1.00 (0.93–1.08)	
> 75th percentile	Ref	Ref		Ref	Ref	
# OB/GYN			0.4963			0.9950
< 25th percentile	0.90 (0.77–1.06)	**0.74 (0.61–0.90)**		0.95 (0.84–1.08)	1.03 (0.94–1.14)	
25-75th percentile	0.96 (0.84–1.09)	**0.76 (0.65–0.89)**		0.95 (0.86–1.05)	1.04 (0.96–1.13)	
> 75th percentile	Ref	Ref		Ref	Ref	
# Hospitals with Ultrasound			0.9655			0.1684
< 25th percentile	1.00 (0.87–1.15)	1.03 (0.87–1.22)		0.93 (0.84–1.03)	1.07 (0.99–1.17)	
25-75th percentile	0.97 (0.87–1.09)	1.00 (0.87–1.15)		0.97 (0.89–1.05)	0.95 (0.88–1.01)	
> 75th percentile	Ref	Ref		Ref	Ref	

Adjusted for county level HCA and SES variables, age, surgery of primary site and seer registry

^a^Additionally adjusted for late stage diagnosis

^b^For interaction terms between age and each HCA variable with late stage diagnosis as outcome

^c^For interaction terms between age and each HCA variable with ovarian cancer mortality as outcome

# Number per million population

Bold indicates significance (*p* value ≤0.05)

## Discussion

Using the population-based SEER registry to analyze data on epithelial ovarian cancer patients and linked county level information on availability of healthcare resources and SES, we observed significant racial differences in the availability of healthcare resources among epithelial ovarian cancer patients in the US. Compared to White patients, Black patients resided in counties with lower health insurance coverage, fewer numbers of oncology hospitals, and fewer numbers of hospitals with ultrasound machines. However, they resided in counties with a higher number of medical doctors and OB/Gyn specialists. We observed that accounting for these differences, in addition to clinical and histologic variables, did not eliminate racial disparities in late stage diagnosis or mortality with epithelial ovarian cancer, even though timely diagnosis and high-quality treatment of epithelial ovarian cancer are most likely dependent on specialty-trained surgeons and healthcare centers with multidisciplinary oncologic care and ultrasound equipment. [27, 28] We observed that among Blacks, residing in counties with fewer medical doctors doubled the odds of late stage diagnosis, however, none of the HCA variables were independently associated with stage at diagnosis for Whites. In contrast, none of the HCA variables independently predicted epithelial ovarian cancer mortality among Blacks, but residing in counties with fewer medical doctors was associated with increased risk of epithelial ovarian cancer mortality among Whites. These results suggest that access to healthcare is a much more complex issue beyond only availability of resources, and racial differences may exist in terms of other aspects of access to care, e.g., accessibility, availability, affordability, accommodation and acceptability, with potentially significant consequences for epithelial ovarian cancer outcomes as well as racial disparities.

Ovarian cancer is one of the most lethal gynecologic cancers [2] with 5-year survival rates of less than 50% after initial diagnosis. [4] Early diagnosis when the tumor is localized to the ovaries is the most important prognostic factor in treatment planning and improvement of survival of these patients. [4, 29] However, early diagnosis of ovarian cancer remains difficult as the disease is often asymptomatic [30] or presents with non-specific symptoms such as back pain, fatigue, constipation or abdominal pain/bloating, which are rarely gynecologic in nature. [31–33] Routine screening for ovarian cancer is currently not recommended except for high-risk groups of women with a genetic predisposition or family history of ovarian or breast cancer. [34] However, a study by Smith et al. using the Medicare linked California SEER database showed that women ages 68 years and older with recent diagnosis of ovarian cancer were more likely to have received clinical evaluations for non-specific abdominal symptoms 6 months before the diagnosis. [35] As ovarian cancer progresses very rapidly [36], this window may be especially crucial in early detection of ovarian cancer. Despite the current lack of highly sensitive screening approaches, improved access to healthcare resources is still a critical factor in early diagnosis and timely and accurate treatment of ovarian cancer, as specialized physicians and ultrasound imaging will be required for definitive diagnosis, timely treatment and improved outcomes.

Differential access to high quality healthcare remains a significant public health issue and contributes to sustained racial differences in cancer outcomes. Other studies have shown that multiple factors beyond individual health insurance status contribute to healthcare accessibility and utilization, including factors relating to quantity, quality, distance, as well as cultural attitudes towards healthcare. [37–40] Tracey et al. showed that that women with ovarian cancer treated in public general hospitals had higher mortality rates compared to those who were treated at a gynecological oncology service, and that distance from the specialized hospital played an important role in the decision making process for where to receive treatment. [41] Other studies have also demonstrated persistent racial disparities in equal access settings such as the VA system and other population based cohorts like Kaiser Permanente-Research on Ovarian Cancer Survival (KP-ROCS) Cohort Study, with Blacks less likely to receive cancer treatment and more likely to die due to their disease compared with other racial groups. [17, 42] In the current study, we found that epithelial ovarian cancer mortality in Blacks was significantly higher than in Whites, consistent with previous studies. [13, 43] However, we did not find significant associations between any HCA variable and epithelial ovarian cancer mortality among Blacks, although reduced number of medical personnel at the county level was associated with higher mortality among Whites. This suggests that interventions to increase availability of medical doctors may help to improve epithelial ovarian cancer outcomes among Whites, while more work is needed to identify factors that mediate the association between availability of healthcare resources, utilization of such resources and epithelial ovarian cancer outcomes among Blacks.

The strength of the study is the use of the population-based SEER registry to assess epithelial ovarian cancer outcomes and examination of county-level availability of healthcare resources and socio-economic status among Black and White women, the two racial groups with the largest disparity. The analysis is strengthened by the use of multi-level modeling to account for correlations between individuals residing in the same county. The analysis focused on the most common subtype of ovarian cancer- epithelial ovarian cancer- which accounted for 86% of cases in Blacks and 90% of cases in Whites, and we observed consistent results when additionally adjusted for histology. There are also several limitations in this study. First, the use of a county as a geographic unit, which is a relatively large geographical area may have resulted in residual confounding if county availability of health care resources does not fully capture the variation in HCA available to individuals. However, analysis of major community resources such as hospitals usually requires larger geographic units of analysis, as they are primarily considered at the municipal, county or state level, and considering smaller geographic areas would have resulted in a large number of geographic regions with missing data. Second, we were unable to adjust for individual level SES variables since these are not present in the SEER database, and instead included area level SES in our models. Further, SEER does not include data on comorbidities, which precluded us from accounting for differential health status of patients at diagnosis which may impact mortality. Lastly, the SEER registry does not provide detailed data on adjuvant chemotherapy or detailed information on timing of surgery, and we also lacked data on the presence of residual disease following debulking or cytoreductive surgery. Due to these limitations, we were unable to account for potential racial differences in the quality of actual treatment received, another important factor for survival although we were able to adjust for the receipt of site-specific surgery.

## Conclusion

In conclusion, this study provides evidence that racial/ethnic disparities exist in the availability of healthcare resources among epithelial ovarian cancer patients, and these influence stage of diagnosis and survival. For Black patients, more research is needed to better understand the factors predicting utilization of existing healthcare resources, and if these influence healthcare outcomes. Further research on other dimensions of healthcare access such as acceptability, accessibility and affordability may inform efforts to increase utilization of existing resources among Black and White cancer patients, eliminating the persistent racial disparity in late stage diagnosis, and ultimately improving survival for all patients.

## Abbreviations

AHRF: Area Health Resource File
ANOVA: Analysis of variance test
HCA: Healthcare access
NH: Non-Hispanic
OB/GYN: Obstetricians/Gynecologists
SEER: Surveillance, Epidemiology and End Results
SES: Socio-economic status
VA: Veteran Administration
